# Transcriptome analysis of hexaploid hulless oat in response to salinity stress

**DOI:** 10.1371/journal.pone.0171451

**Published:** 2017-02-13

**Authors:** Bin Wu, Yani Hu, Pengjie Huo, Qian Zhang, Xin Chen, Zongwen Zhang

**Affiliations:** Institute of Crop Science, Chinese Academy of Agricultural Sciences (CAAS), Beijing, China; University of Western Sydney, AUSTRALIA

## Abstract

**Background:**

Oat is a cereal crop of global importance used for food, feed, and forage. Understanding salinity stress tolerance mechanisms in plants is an important step towards generating crop varieties that can cope with environmental stresses. To date, little is known about the salt tolerance of oat at the molecular level. To better understand the molecular mechanisms underlying salt tolerance in oat, we investigated the transcriptomes of control and salt-treated oat using RNA-Seq.

**Results:**

Using Illumina HiSeq 4000 platform, we generated 72,291,032 and 356,891,432 reads from non-stressed control and salt-stressed oat, respectively. Assembly of 64 Gb raw sequence data yielded 128,414 putative unique transcripts with an average length of 1,189 bp. Analysis of the assembled unigenes from the salt stressed and control libraries indicated that about 65,000 unigenes were differentially expressed at different stages. Functional annotation showed that ABC transporters, plant hormone signal transduction, plant-pathogen interactions, starch and sucrose metabolism, arginine and proline metabolism, and other secondary metabolite pathways were enriched under salt stress. Based on the RPKM values of assembled unigenes, 24 differentially expressed genes under salt stress were selected for quantitative RT-PCR validation, which successfully confirmed the results of RNA-Seq. Furthermore, we identified 18,039 simple sequence repeats, which may help further elucidate salt tolerance mechanisms in oat.

**Conclusions:**

Our global survey of transcriptome profiles of oat plants in response to salt stress provides useful insights into the molecular mechanisms underlying salt tolerance in this crop. These findings also represent a rich resource for further analysis of salt tolerance and for breeding oat with improved salt tolerance through the use of salt-related genes.

## Introduction

Soil salinity is one of the most important abiotic stresses and is a limiting factor for plant production worldwide [1]. To date, approximately 7% of total land area (1,000 million ha) and 20% of the irrigated arable land in arid and semi-arid regions is salt affected, and this number is increasing [2, 3]. Salinization of agricultural soils causes ion imbalance and hyperosmotic stress in crops, leading to reduced crop growth, yields, and productivity. Sodium (Na^+^) and chloride (Cl^-^) are the two key ions responsible for both osmotic and ion-specific damage, which significantly reduce crop growth and yields [4]. However, such soil may be utilized either after reclamation or by growing salt-tolerant plant species. Therefore, efforts have been undertaken to increase the salt tolerance of plants by traditional breeding as well as biotechnological approaches [5].

Oat (*Avena* Linn.) is an important cereal crop that is grown worldwide for fodder as well as grain. Oat is one of the oldest crops and is still widely cultivated worldwide, including at high latitudes and in restrictive climates. Oat is highly nutritious for human and cattle consumption. Knowledge of the importance of oat in human nutrition is increasing following reports of the cholesterol-lowering, antioxidant, and other health-related properties of oat and oat products and components [6, 7]. The *Avena* genus includes some species referred to as naked oat, whose seeds are not as tightly husked as the others. Among these species, *Avena chinensis* (Fisch. ex Roem. & Schult) Metzg. is a dominant crop and traditional food for local people in some marginal areas of north China affected by salinity and aridity, thus playing an important role in the local economy and ecological environment [8]. Compared with wheat, soybean, cotton, and other seasonal crops, oat has much higher sodium ion levels [9–13]. Oat cultivation is even considered to represent a helpful biological measure to improve saline lands due to its high capacity to accumulate salt ions in its straw, which is widely used as forage for livestock [14]. Therefore, tolerance to abiotic stresses such as salt and drought is a highly important trait in oat breeding. However, abiotic stress tolerance is a quantitative trait controlled by multiple genes [15–16]. To improve abiotic stress tolerance in oat, it is essential to understand the fundamental molecular mechanisms behind the stress tolerance of this crop.

Compared with other plants, little is known about the molecular mechanisms underlying oat stress tolerance, which is largely due to oat’s complex, large genome, as well as limited funding for oat research. Cultivated oat is an allohexaploid (2n = 6x = 42) that was derived from three ancestral diploid Avena genomes (A, C, and D), whose complexity is associated with its large and repetitive genome. Bennett found that the size of the hexaploid oat genome is 1C = 11.7 pg, which corresponds to 11,443 Mbp (1 pg = 978 Mbp) [17]. The development of Next Generation Sequencing (NGS) technologies has a provided a low-cost way to generate genomic resources on a large scale [18]. Elucidating the molecular basis of abiotic stress tolerance in crops is becoming more easily achievable using these approaches [19]. In oat, pioneering work has already begun to uncover large portions of the transcriptome of immature seeds, which greatly increases the number of EST sequences available for analysis of genes related to nutrition [20]. However, to date, genome-wide expression profiling of oat under salt stress has not previously been reported.

To gain a global view of transcriptional changes in oat under salt stress, we performed a transcriptome study of oat at different stages of salt treatment versus the control using NGS. Through bioinformatic exploration and further quantitative Reverse-Transcription PCR (qRT-PCR) validation of some representative transcripts, we comparatively analyzed and functionally annotated a subset of transcripts in salt-treated oat. The dynamic transcriptome expression profiles of salt-stressed oat obtained in this study provide useful insights for further analyses of the salinity tolerance mechanism in oat. Moreover, the genes found to be differentially expressed under salt stress in this study may facilitate the identification of key genes representing suitable targets for biotechnological manipulation with the aim of improving salinity tolerance in oat.

## Materials and methods

### Plant materials and growth conditions

The highly salt tolerant oat cultivar ‘*HanYou-5*’ was identified from 278 naked oat accessions collected from different ecological regions in an earlier experiment [21]. Sterilized seeds were germinated for 5 days at room temperature on moist filter paper before being transferred to Hoagland nutrient solution under a 16 h light/ 8 h dark cycle, day/night temperatures of 25°C/20°C, and relative humidity of 70%. Salt treatment was carried out as follows: when the third leaves emerged, oat seedlings were treated with 100 mM NaCl dissolved in Hoagland nutrient solution; those without NaCl treatment were used as the control. Whole plant samples were harvested at 2, 4, 8, 12, and 24 h after salt stress treatment and immediately frozen in liquid nitrogen for gene expression analysis. The control and each treatment were biologically and temporally repeated in three independent and parallel experiments to avoid sampling errors.

### Measurements of physiological variables

The physiological assays were carried out according to the methods in Sunkar with minor modifications [22]. For the calculation of relative water content (RWC), plant fresh weights (FW) were measured immediately after harvest, and the plants were then floated on deionized water for 8 h at 4°C. The turgid plants were quickly weighed (TW) and their dry mass (DW) was measured after oven-drying at 105°C for 10 min followed by 80°C for 24 h. RWC was calculated as follows: RWC (%) = (FW− DW)/(TW−DW)×100. Free proline content was determined according to colorimetric method. Briefly, 0.2g oat plant powder which was grinded by using a mortar and pestle was homogenized in 5% sulpho salicylic acid. After extraction, an equal volume of glacial acetic acid and ninhydrine reagent were added to the supernatant and the mixture was boiled in a water bath for 1 h and then cooled in an ice bath. The solution was partitioned against 2 ml of toluene and absorbance at 520 nm measured in this organic layer. A calibration curve was performed using commercial proline as a standard. Soluble sugar concentration was measured by the anthrone method using glucose as standard. In brief, 1 ml of anthrone reagent (0.2% v/v anthrone on 95% sulfuric acid) was added to the extract, heated with boiling water for 15min, and the absorbance at 630nm was measured using a UV-vis spectrophotometer (Shimadzu, UV-2550). For inorganic cation detection, the harvested samples were immediately washed with distilled water to remove the nutrient solution on the surface. The samples were then dried and baked to ash. Cations contained in the ground ash samples were extracted in Milli-Q water with vigorous shaking for 24 h. After brief centrifugation, the supernatants were filtered using cellulose acetate filters and the concentrations of Na^+^, K^+^, and Ca^2+^ were measured using an ion chromatograph system (SHIM-PACK IC-C3; Shimadzu, Kyoto, Japan). To ensure more accurate measurements, >5 independent plants were pooled in a single sample to reduce the effects of biological variation. Superoxide dismutase activity was assayed using a modified NBT method. Tissue samples (0.2 g) were homogenized in 1.2 mL of 0.2 M potassium phosphate buffer (pH 7.8 containing 0.1 mM EDTA). The assay mixture (2 ml) contained 50 mM phosphate buffer, pH 7.8, 9.9 mM L-methionine, 55 mM NBT, and 0.025% (w/v) Triton X-100. Absorbance of the samples was measured at 560 nm and the enzyme activity of a sample was determined from a standard curve obtained using pure SOD. Catalase activity was measured by monitoring the decomposition of H_2_O_2_ by measuring the decrease in absorbance at 240 nm of a reaction mixture consisting of 50 mM potassium phosphate buffer (pH 7.0), 10 mM H_2_O_2_ and enzyme extract. The extinction coefficient of H_2_O_2_ (40 mM^−1^ cm^−1^ at 240 nm) was used to calculate the enzyme activity that was expressed in terms of millimoles of H_2_O_2_ per minute per gram fresh weight). Peroxidase activity was measured based on the change in absorbance at 470 nm due to the oxidation of guaiacol. Enzyme activity was expressed in terms of μmol of guaiacol oxidized min^−1^ g^−1^ fresh weight at 25°C. All the experiments were conducted in triplicate (n = 3) and statistical analysis of the data from the control and the salt-stressed plants was performed by analysis of variance (One-Way ANOVA), using SPSS 16.0 software. Data are presented as the means and standard deviations (SD) of three replicates.

### RNA extraction, cDNA library preparation, and GS-FLX pyrosequencing

Total RNA from the control and NaCl-treated samples was extracted with an RNeasy Plant Mini kit (Qiagen) following the manufacturer’s instructions. After digestion with RNase-free DNase I (Promega) to eliminate DNA, Poly (A) mRNA was isolated from total RNA using an Oligotex mRNA mini kit (Qiagen). mRNA extracted from the different stages of salt-stressed oat seedlings were prepared for cDNAs synthesis separately, and the same amount of mRNA from unstressed plants was used for the control. The quality of total RNA, mRNA, and final double-stranded cDNA samples was verified with a NanoDrop (Thermo Scientific, Waltham, USA) and Agilent 2100 Bioanalyzer (Agilent Technologies). Approximately 5 μg of each cDNA sample was sheared via nebulization into small fragments and sequenced using the Illumina HiSeq 4000 platform. The transcriptome datasets are available at the NCBI Sequence Read Archive (SRA) under accession number SRR5097544- SRR5097549.

### RNA-seq data processing and de novo assembly

Raw data were pre-processed using est2assembly [23] to remove adaptor sequences, ribosomal RNA, and other contaminants that may interfere with clustering and assembly. After screening and trimming, the final high quality reads were assembled using Trinity with default parameters [24].

### Functional annotation and digital expression analyses

Functional annotation of the assemblies was performed by sequence similarity searches using the BLAST program against the NCBI nucleotide sequence database (nt), non-redundant protein sequence database (nr), and UniProt (Swiss-Prot) with e-value threshold of 10^−5^ [25]. Gene ontology (GO) classification was performed by Blast2GO [26] via GO id annotated using Perl and R programs. The unigene sequences were also aligned to the COG database to predict and classify their functions. KEGG pathway information was assigned to the unigene sequences using the KAAS-KEGG Automatic Annotation Server online [27].

For differentially expressed gene (DEG) analysis of salt-stressed oat, the clean reads of each library were mapped to the sequences of each unigene, and the reads per kilobase of transcripts per million mapped reads (RPKM) values were calculated via RNA-Seq analyses [28]. The significance of differences in gene expression under salt stress was determined using DEGseq, an R package [29]. False discovery rate (FDR) was applied to identify the threshold of the P value in multiple tests [30]. When FDR was less than 0.05 and log2 ratio was greater than 1 (two-fold change) between two conditions, the unigenes were considered to be differentially expressed.

### SSR detection

Simple sequence repeats (SSRs) were identified using MISA [31] and filtered to represent unique polymorphisms using custom scripts. The minimum number of nucleotide repeats required was 10 for mononucleotide repeats, seven for dinucleotide repeats, and five for other repeats, that is, tri-, tetra-, penta-, and hexanucleotide repeats, and the maximum number of bases interrupting two SSRs in a compound SSR was set to 100 bp. Primer design was performed in batch with Primer3 [32] using default parameters and Perl scripts.

### Confirmation of expression profiles by quantitative RT-PCR

Differences in the expression levels of oat transcripts detected by RPKM analysis under salt stress were confirmed by relative quantitative real-time RT-PCR. The RNA used for qRT-PCR was prepared independently from that used in RNA sequencing. First, total RNA isolated from oat plants at various stages of salt stress was digested with RNase free DNase I (Promega) at 37°C for 15 min and phenol extracted to remove genomic DNA contamination. Then, 1 μg purified total RNA from different stages was reverse transcribed into cDNA using oligo d(T)15 primers (Promega) and SuperScript III reverse transcriptase (Life Technologies). The synthesized cDNA was diluted to 200 μl in sterile water for qRT-PCR analysis. For qRT-PCR, a Platinum SYBR Green qPCR SuperMix-UDG kit (Life Technologies) was used according to the manufacturer’s recommendations on an ABI 7500 Real-Time PCR System (Applied Biosystems Foster City, CA, USA). The cDNAs from three independent RNA samples per group were synthesized, and three replicates per sample were carried out for qRT-PCR validation. The cycling conditions were as follows: 95°C for 10 min, followed by 40 cycles of 95°C for 15 s and 60°C for 1 min. Twenty four unigenes that were assumed to be differentially expressed according to RPKM value were chosen for qRT-PCR validation; their specific primers are listed in additional file 1. The relative expression level of each unigene was calculated by the 2^-ΔΔCT^ method using the actin gene as the internal control [33]. The specificity of amplification was verified at the end of each PCR run using 7500-system SDS Dissociation Curve Analysis Software.

## Results

### Physiological changes of oat under salt stress

**Fig 1:**
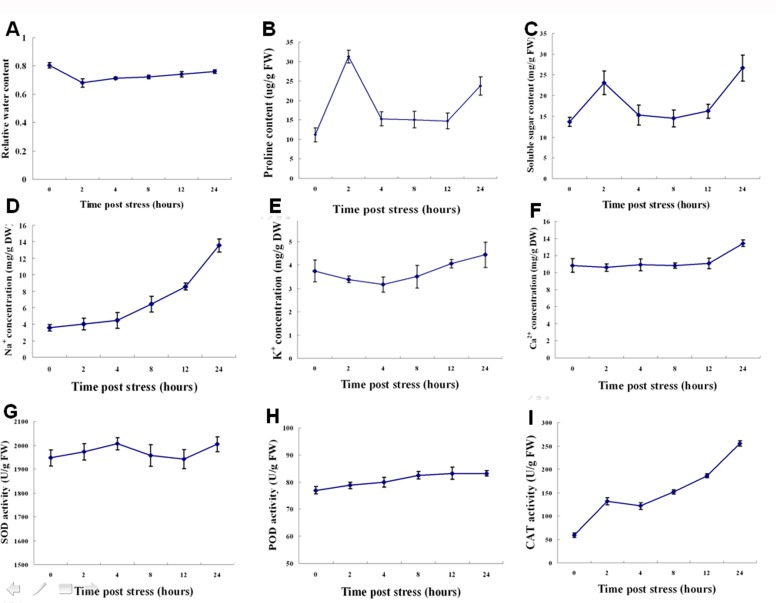
Physiological changes of oats in response to various durations of salt stress. (A) Relative water content (RWC) levels; (B) Proline content; (C) Soluble sugar content; (D) Na^+^ concentration; (E) K^+^ concentration; (F) Ca^2+^ concentration; (G) Superoxide dismutase (SOD) activity; (H) Peroxidase (POD) activity; (I) Catalase (CAT) activity in seedlings treated with 100 mM NaCl. Data represent means ± SE of three independent experiments.

Since salt stress directly affects osmotic potential in plant tissues and induced the active oxygen damage to plants, we investigated the change patterns of osmotic regulator and activities of the protective enzymes systems such as SOD, POD, and CAT in oat plant during the first 24h under stress. Results of all collected samples were shown in Fig 1. From the figure we can find out that different physiological indexes showed different patterns of change. RWC decreased immediately after the onset of stress while significant increases in proline and soluble sugar and the contents reached the peak after 2h then dismounted rapidly afterwards, and after 12h salt stress treatment, the soluble sugar and proline contents stopped falling and began to rise. It should be noted that, under salt stress conditions, the contents of soluble sugar and proline were still higher than that of the control even at the lowest level. As for ion concentration, The Na^+^ concentration increased immediately after salt stress and the rate of rise is accelerated after 4h, while K^+^ concentration declined firstly and began to rise after 4h. The change of Ca^2+^ concentration is relative small at the beginning of salt stress and began to rise after 12 hours. ROS scavenging and detoxifying system, the activity of CAT increased significantly at the beginning of salt stress, after dropped a little at 4h, and then began to rise again. Similar trends were also found in the changes of SOD activity, but the emergence of activity decreased more lately (4h) and lasted longer (up to 12h), while the activity of POD enzyme increased stably but the extent of increasing is relative smaller (about 8.25% at 24h). These results indicate the complexity of oat physiological changes in response to salt stress and these changes showed certain time order.

### Sequencing and de novo assembly of the oat transcriptome

**Fig 2:**
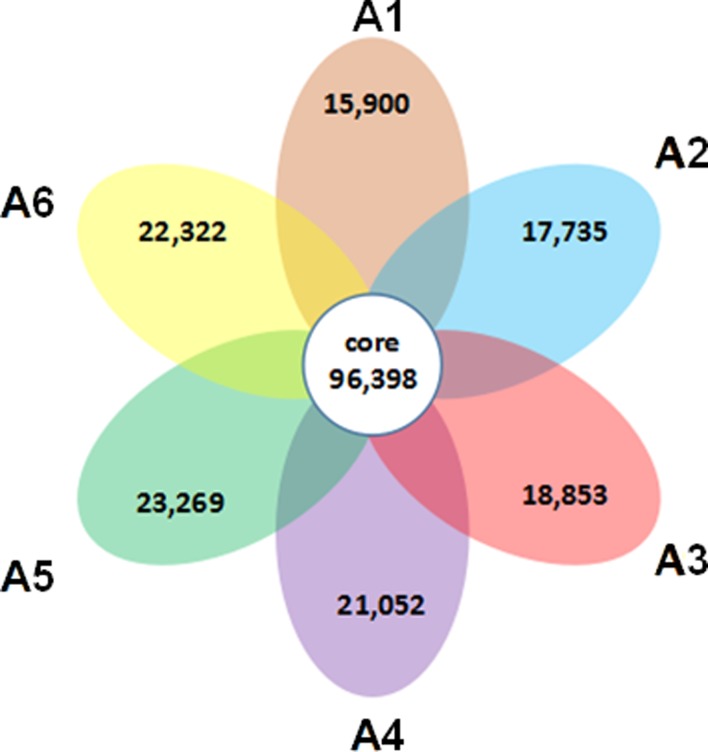
Flower plot, the gene expression statistics of different conditions. Flower plot showing overlaps among differential expressed genes under salt stress in oat. Different group indicate the detected genes under normal growth conditions (A1), and 2 (A2), 4 (A3), 8 (A4), 12 (A5), and 24 (A6) hours of salt stress treatment. Numbers in one circle denote salt stress-specific genes and numbers in intersecting circles denote overlapped genes.

Six libraries were prepared from whole plants of cultivated hulless oat (*Avena chinensis* [Fisch. ex Roem. & Schult] Metzg._ cv. ‘HanYou-5’) under normal and salt-stressed conditions, respectively. To obtain a wide range of salt stress-responsive oat transcripts, five independent RNA samples from oat plants sampled at 2, 4, 8, 12, and 24 h after salt stress treatment were used for synthesis of the salt-stressed cDNA library. These cDNA libraries were then sequenced on Illumina HiSeq 4000 platform, generating a total of 72,291,032 and 356,891,432 raw reads in the control and salt-treated samples, respectively. After filtering out adaptor sequences, low quality reads, and ambiguous reads, 63,367,896 and 319,815,896 high quality reads were obtained in the control and salt-treated samples. Differential expressed transcripts under salt stress were shown in Fig 2. Among them, about 75.07% (96,398/128,414) unigenes was shared by all groups. To obtain more complete and representative information about the *A*. *chinensis* transcriptome, all cleaned reads were merged to form a combined dataset. A total of 128,414 putative uingenes were obtained from the combined database, with the average length of 1,189 bp. An overview of the sequencing and assembly data is presented in S1 Table. All assembled unigenes sequences obtained in this study can be accessed as a fasta file (additional file 2).

### Annotation of the putative unigenes and their functional categorization

The de novo assembled oat unigenes were annotated through homology searches against nucleotide and various protein databases with an e-value cutoff of 10^−5^. Of the 128,414 unigenes, 87,784, 85,896, and 64,323 had significant hits in the GenBank nucleotide sequence database (nt), non-redundant protein sequence database (nr), and Swiss-Prot database, respectively. Based on homology with sequences of different species, the majority of hits (28.2%) were found against stiff brome (*Brachypodium distachyon*), followed by winter barley (*Hordeum sativum*; 20.9%), barbed goatgrass (*Aegilops squarrosa*; 14.0%), and red wild einkorn (*Triticum urartu*; 8.3%). The high sequence homology of our identified transcripts to those of other graminaceous crops helps confirm the reliability of our RNA-seq dataset.

All putative unigenes were assigned GO classifications of their molecular functions to systematically characterize the transcriptome data. Among the 128,414 uingenes, 70,511 were assigned to different GO terms, including77,246 transcripts assigned to GO terms in the molecular function category, 181,717 in the cellular component category, and 197,926 in the biological process category (additional file 3). In the domain “biological process”, unigenes related to “metabolic processes” (22.22%) and “cellular processes” (20.88%) were the most abundant classes, while in the domain “cellular component”, “cell” and “cell parts” (45.35%) were predominant. Under the “molecular function” category, the majority of unigenes were classified in the “catalytic activities” (43.65%) and “binding” (42.79%) categories.

COG classification of whole transcripts revealed that the unigenes were clustered into 25 functional categories (additional file 4). “General function prediction only” (12.31%) was the major COG category, followed by “Transcription” (9.02%) and “Translation, ribosomal structure and biogenesis s” (8.89%). “Nuclear structure” (0.01%) was the smallest group. Using KEGG (Kyoto Encyclopedia of Genes and Genomes) analysis, all unigenes were assigned to 128 pathways (additional file 5). The major pathways were metabolic pathways (29.64%), RNA transport (17.96%), and mRNA surveillance pathway (15.59%). Compared to the control, ABC transporters, arginine and proline metabolism, vasopressin-regulated water reabsorption, brassinosteroid biosynthesis, and other secondary metabolite pathways were induced under salt stress. KEGG pathway map shows that, in the category “ABC transporters”, a number of genes for ion transport exhibited higher transcript abundance under salt stress than under normal conditions. Further analyses indicated upregulation of transcripts for ABC transporter genes involved in ABC B/C/D/G and other putative transporters in salt-stressed oat (additional file 6).

### Identification of transcription factor genes in the salt stressed transcriptome

All assembled unigenes were aligned against the Plant Transcription Factor Database (PlnTFDB; version 3.0; http://plntfdb.bio.unipotsdam.de/v3.0/) by Blastx with e-values below 10^−5^ and identity of over 70% [34]. A total of 5,318 unigenes were found in the assembled uingenes and more than half of them (2,897) were found differentially expressed at one of the detected salt stress stage. The most highly represented TF genes belonged to the MYB and MYB_related, bHLH, WRKY, and NAC families of TFs, members of which function in abiotic stress responses [35]. A complete list of TF genes and their respective TF families is presented in additional file 7.

### Identification of salt-responsive transcripts and their functional annotation

We performed differential expression analysis of oat genes by calculating the RPKM value of each unigenes. Comparison of the RPKM values between normal growth and salt stress conditions showed that the expression levels of 65,009 putative unigenes were significantly (>2-fold, FDR < 0.05) altered at various salt stress stage. These genes include salt sensor or signal transduction genes, salt responsive TF genes, plasma membrane stabilization-related genes, osmosensing-responsive genes, and genes encoding detoxification enzymes (additional file 8).

To precisely identify the biological processes specifically functioning in oat under salt stress, ESTs with significant expression differences were further annotated with GO functional annotations. Compared with GO annotation of the total set of unigenes, GO assignment of DEGs resulted in a somewhat different distribution of GO terms (additional file 9). For example, in the GO domain “biological process”, more salt-responsive DEGs were assigned to the categories “signaling” and “signaling process” compared to unigenes under control condition, whereas fewer of these DEGs were assigned to “growth” and “metabolic process”. The most noticeable differences in the GO domain “molecular function”, were an increase in salt-responsive DEGs in the categories “extracellular region” and a decrease in those in “macromolecular complex”. Some of the overrepresented categories include “protein binding transcription factor activity”, “transcription regulator activity”, and “transporter activity”.

### Identification and characterization of SSRs

SSRs markers are widely used in genetic and population studies. However, the limited availability of SSR markers for oat has hampered many areas of oat research. Compared with SSRs identified from total genomic DNA, SSRs identified from the transcribed regions of the genome are more likely to be linked to loci that contribute to agronomic traits [36]. To develop additional markers for molecular breeding of oat, we scanned the assembled contigs for SSR markers. A total of 18,039 SSRs were found. Primers targeting the SSRs and the expected lengths of amplified fragments are listed in additional file 10. SSRs in the transcripts were detected at a frequency of approximately one SSR per 8.46 kb. Analysis of these SSR motifs revealed that the distribution of SSR repeats was uneven. Most of the simple repeats were trinucleotide repeats (10,337; 57.30%), followed by dinucleotide repeats (4,307; 23.88%), mononucleotide repeats (1657; 9.19%), and tetranucleotide repeats (645; 3.58%). These newly identified SSRs represent useful tools for marker-assisted selection and breeding programs in oat.

### Validation of DEGs by qRT-PCR

**Fig 3:**
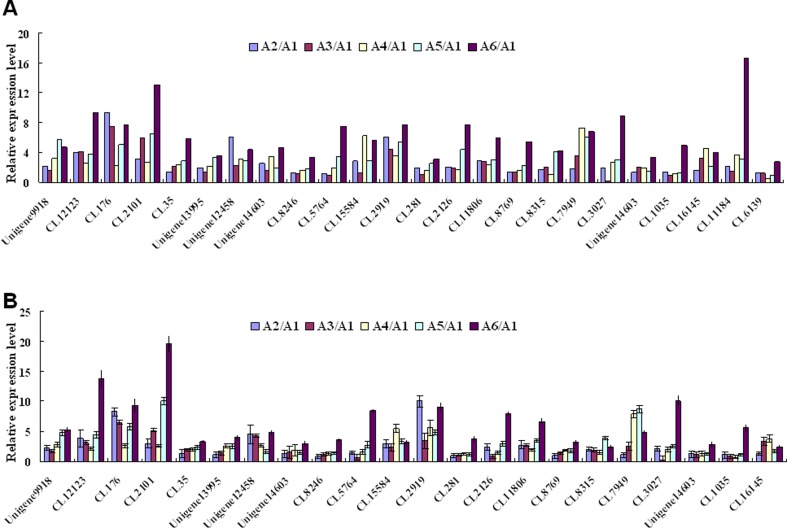
Expression pattern confirmation of selected genes by qRT-PCR. (A) Changes in the relative expression levels of 24 selected genes as determined by RNA-Seq. (B) Changes in the relative expression levels of 24 selected genes as determined by qRT-PCR. The Y axis indicates the relative fold-change in transcript abundance under conditions of salt stress in relation to the control. Abbreviations indicate ‘HanYou-5HanYou-5’ under normal growth conditions (A 1), and 2 (A 2), 4 (A 3), 8 (A 4), 12 (A 5), and 24 (A 6) hours of salt stress treatment. qRT-PCR results represent mean ± standard deviation (SD) of three experimental replicates.

**Fig 4:**
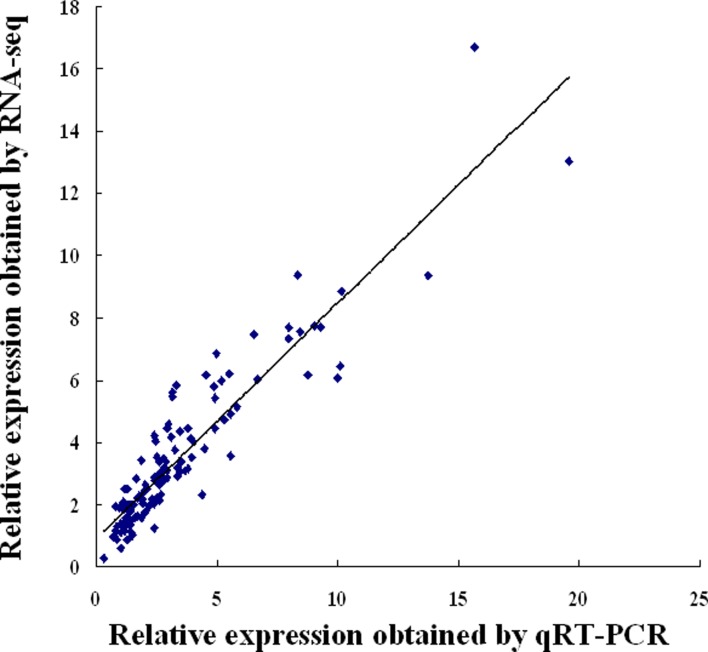
Comparison between the relative expression obtained from RNA-Seq data and qRT-PCR. Correlation analysis of 24 highly differentially expressed genes under salt stress based on qRT-PCR and RNA-seq data; Pearson correlation coefficient (r) is 0.9235 (P < 0.05).

To further validate the NGS results, we conducted qRT-PCR analysis of selected metabolic pathway-related DEGs. The qRT-PCR results are strongly correlated with the DEG data. Although the exact change did not exactly match each other, the expression trends of all 24 genes from qRT-PCR and RNA-seq analyses were largely consistent (Pearson’s correlation coefficient r = 0.92), demonstrating the reliability of the RNA-seq results (Fig 3) and the correlation analysis of DEGs under salt stress based on qPCR and RNA-seq data was shown in Fig 4.

## Discussion

### Functional annotation and classification of assembled unigenes

Salt tolerance is a complex quantitative trait that is regulated by a large number of genes. Understanding the plant response to salt stress requires a global analysis of stress-responsive genes. NGS represents a valuable tool for obtaining a nearly complete characterization of transcriptomic events occurring at a specific stage. Due to its large, complex genome, oat is one of the least explored cereal grain crops in terms of genomic and transcriptomic research and few such studies have been performed in cultivated oat. In this study, we characterized the salt stress response of a salt-tolerant oat cultivar at the transcriptomic level using large-scale sequencing data. We identified 128,414 putative unique transcripts reflecting general transcriptomic responses to salt stress and uncovered a collection of salt-stress-regulated gene networks, providing a basis for further studies.

Gene annotation is an important step in NGS data analysis in which biological information is attached to predicted genes or unigenes. The presence of a high proportion of unigenes with high similarity to protein sequences from other plant species helps confirm the integrity of transcript sequences assembled from NGS data [37]. In the current study, 66.89% unigenes matched at least one homolog in the NR database, as determined by BLAST and functional bioinformatics analyses, which helps confirm the reliability of the assembled oat unigenes and suggests that these sequences can be used for further investigations. GO analysis provides a standardized set of terms to describe genes and gene products consistently across different species and databases [38]. GO analysis is widely used for annotation and for comparing gene products of different species to gain general insights into the biological roles of all genes revealed by high-throughput analysis [39]. In this study, based on GO analysis of DEGs, several enriched biological processes and metabolic pathways were identified, providing insights into the possible molecular mechanism underlying the salt stress response in oat. For example, many DEGs were enriched in GO terms such as “transporter activity”, “protein binding transcription factor activity”, and “response to stimulus”. This information will be useful for elucidating salt tolerance mechanisms and for finding new salt-stress-related genes specific to *A*. *chinensis*. KEGG enrichment analysis revealed significantly enriched metabolic pathways and signal transduction pathways involving the DEGs. These upregulated pathways include ABC transporters, arginine and proline metabolism, vasopressin-regulated water reabsorption, MAPK signaling pathway, Calcium signaling pathway, peroxisome and plant hormone signal transduction, and other secondary metabolite pathways, suggesting that maintaining osmotic balance and membrane integrity play vital roles in salt stress tolerance in oat. Proline plays an important role in osmotic and salinity stress responses in higher plants. In this study, genes involved in salt-stress tolerance pathways, such as those involved in arginine and proline metabolism, were found to be induced under stress. Measurement of physiological responses showed that the proline content increased significantly (from 11.21 to 31.28 μg/g FW) undersalt stress conditions, which helps confirm the reliability of our DEG analysis results.

### Transcription factors and their roles in the oat salt stress response

TFs are viewed as molecular switches that link signal transduction pathways to gene expression. In this study, 5,318 TF genes were identified in the assembled transcripts and differential expression analysis showed that most of them were differentially regulated under salt stress. These results also imply that TFs play important roles in modulating the acclimation response of oat to salt stress. Among these TFs, MYB and MYB-related TF family were most abundant, followed by those in the bHLH, WRKY, and NAC TF families.

The MYB family is one of the largest TF families in plants. In the current study, transcriptomic analysis revealed that approximately 10% of differentially expressed TF genes were in the MYB and MYB-related TF family. Some of MYB family genes show high expression level at salt stress early stage. For example, the MYB TF gene CL7651 (homologous to TaMYB1) is significantly up-regulated at salt stress early stage (2h), after a little decreasing (4h) then increasing continually at the detected salt stress stage. Previous study also shows that *MYB1* gene was induced at salt stress early stage and regulated a lot of stress response genes. In potato, a cDNA encoding MYB-like gene named *StMYB1R-1* was identified as drought stress response gene. Over expression of *StMYB1R-1* enhanced the expression of drought regulated genes such as *AtHB-7*, *RD28*, *ALDH22a1*, and *ERD1*-like, indicating that StMYB1R-1 functions as a transcription factor involved in the activation of drought-related genes. Further analysis shows that the transcript level of StMYB1R-1 were enhanced in response to several environmental stresses in addition to drought stress but were unaffected by biotic stresses. In particular, northern blot analysis demonstrated that *StMYB1R-1* was induced at salt stress early stage [40]. These results indicate that some MYB and MYB-related TFs play important roles in the early salt stress response of oat.

NAC proteins are plant-specific transcription factors and have various functions not only in plant development but also in abiotic stress responses. Stress-inducible NAC genes have been shown to be involved in abiotic stress tolerance. Transgenic Arabidopsis and rice plants overexpressing stress-responsive NAC genes have exhibited improved drought tolerance. Some of the field tested transgenic plants have indeed performed better under stress conditions [41]. In our study, about 300 NAC family genes were detected. Some of these genes (Unigene13995, Unigene13991, CL2541, CL5374, CL14700) steadily up-regulated during the detected salt stress stages indicating their overexpression maybe related to oat long-term stress tolerance mechanism.

Other types of TFs, such as bHLH, bZIP, C2H2, and WRKY, were also found to be responsive to salt treatment in oat. These TF families, which have not been previously characterized in oat, are involved in a variety of processes and play important roles in responses to abiotic and biotic stress in model plants [42]. The discovery of the involvement of various TF families and different members of these families in the oat salt response indicates that a complicated transcriptional regulatory network is involved in this response. The identification of these TFs will be useful for studying the transcriptional regulatory switches involved in the adaptation of oat to environmental stress.

### Osmotic adjustment is important for salt tolerance in *A*. *chinensis*

Maintaining and re-establishing homeostasis in the cytoplasm, which usually relies on the reorganization and spatial distribution of many key metabolites, is crucial for proper metabolic responses of plants under salt stress; this process requires rapid synthesis of osmolytes and efficient transport machinery [43]. Measurement of physiological changes shows the content of some osmolytes, such as proline and soluble sugar, up-regulated significantly at the salt stress early stage indicating that oat has a high capacity for osmotic adjustment. On the other hand, transcriptomic analysis revealed that approximately 8% of DEGs in oat encode membrane transporter-related proteins, including ATP binding cassette (ABC) transporters, ATPase, ion transporter, and aquaporin. The ABC transporter superfamily is a class of ubiquitously distributed proteins whose main function is to mediate the energy-driven transport of a multitude of substances across membranes. ABC transporter genes in *Arabidopsis* display differential responses at the transcriptional level after abiotic and biotic treatments [44]. In the current study, the ABC C type subfamily was the most abundant type identified in oat. The higher expression levels of ABC transporter genes (CL1467, CL2126, CL11006, Unigene4438, Unigene23610, Unigene33051) under salt stress indicates that oat enhanced the transport ability in response to salt stress.

To cope with osmotic imbalance and ion toxicity, once Na^+^ is taken up into the cell, ATPases are induced to provide the driving force for Na+ transport by Na^+^/H^+^ antiporters, which are essential for reestablishing cellular ion homeostasis and for maintaining the balance of electrochemical potential in salt-stressed plants. The increased expression of these ATPase genes indicates that oat has a high capacity for maintaining osmotic balance. Moreover, high H^+^-pumping activity helps maintain high concentrations of K^+^ in salt-stressed oat. Indeed, intracellular K^+^ and Na^+^ homeostasis is important for the activities of many cytosolic enzymes and for maintaining proper cytosolic K^+^ to Na^+^ ratios to reduce the deleterious effects of Na^+^ [45]. The identification of transcripts related to K^+^ uptake suggests that the outstanding K^+^ uptake capacity of oat is probably conferred by its enhanced K^+^ uptake system, which is achieved by the increased expression of K^+^ transporter genes such as CL8769. In addition to these transporter genes, genes encoding other types of transporters, such as magnesium transporter (CL9008), sulfate transporter (CL154), and sugar transporter (CL281), were also upregulated in salt stressed oat. The increased expression of these genes under salt stress may help the plant maintain homoeostasis and overcome saline toxicity, which likely increases salt tolerance in oat. These findings help explain the results of physiological measurement which shows the accumulation of detected ions in the short-term response to salt stress.

## Conclusions

This is the first report of comprehensive transcriptome analysis and identification of DEGs in *A*. *chinensis* under salinity stress based on Illumina HiSeq 4000 platform. This analysis revealed 128,414 unique sequences. Functional analysis showed that salinity stress in oat affects a variety of genes involved in different biological processes, osmotic adjustment, and regulatory networks. In addition, the thousands of SSR markers predicted in our ESTs represent valuable molecular markers that should facilitate genomic studies, such as linkage map construction and genetic mapping of QTLs associated with important agronomic traits in oat. This study increases our knowledge of the salt stress response of *A*. *chinensis* at the molecular level. The data can be used as a reference for further transcript expression studies in oat.

## Supporting information

S1 Table Summary of transcriptome sequencing and assembly results. (XLS)

S1 File List of primers used in RT-PCR. Excel file containing a list of primers used for RT-PCR experimental validation.
(XLS)

S2 File Fasta file containing the 128,414 assembled sequences of oat. Sequences were de novo generated by assembling nearly 0.43 billion filtered, high quality reads.
(RAR)

S3 File Gene ontology analysis of all expressed unigenes. All unigenes with best BLAST hits were aligned to the GO database; 70,511 putative unigenes were assigned to at least one GO term and were grouped into three main GO categories.
(XLS)

S4 File COG classification of all PUTs. A total of 50,864 putative proteins showing significant homology to those in the COG database were functionally classified into 25 molecular families.
(TIF)

S5 File Number of putative unigenes involved in various KEGG pathways. Assignment of 75,725 putative proteins to different KEGG pathways.
(XLS)

S6 File Analysis of pathways related to ABC transporters. Map displays selected steps from KEGG pathways map02010 ‘ABC transporters’. Colors indicate significant expression, respective metabolite content ratios between salt stressed and normal conditions, red indicates higher relative levels in salt stressed samples, green indicates lower relative levels under salt stress.
(TIF)

S7 File Distribution of transcription factor family genes. Complete list of 5,318 putative unigenes identified as TFs. The distribution of their respective TF families is also presented.
(XLSX)

S8 File Significantly differentially expressed putative unigenes under salt stress. PUTs with absolute value of |log2Ratio| > 1 and FDR < 0.05 were identified as DEGs.
(XLSX)

S9 File GO annotation of differentially expressed unigenes under salt stress. GO annotation of DEGs was based on an e-value cutoff of <10^−5^.
(XLS)

S10 File SSRs and primer details. File containing the assembled PU-derived SSR markers for potential use in oat breeding programs. A total of 1,941 SSRs were found within 1,798 different transcripts.
(XLSX)
