# Supplementary figures and images for: Transcriptome analysis of hexaploid hulless oat in response to salinity stress
Source: PLoS One. 2017 Feb 13;12(2):e0171451. doi: 10.1371/journal.pone.0171451 (PMC5305263; doi:10.1371/journal.pone.0171451)

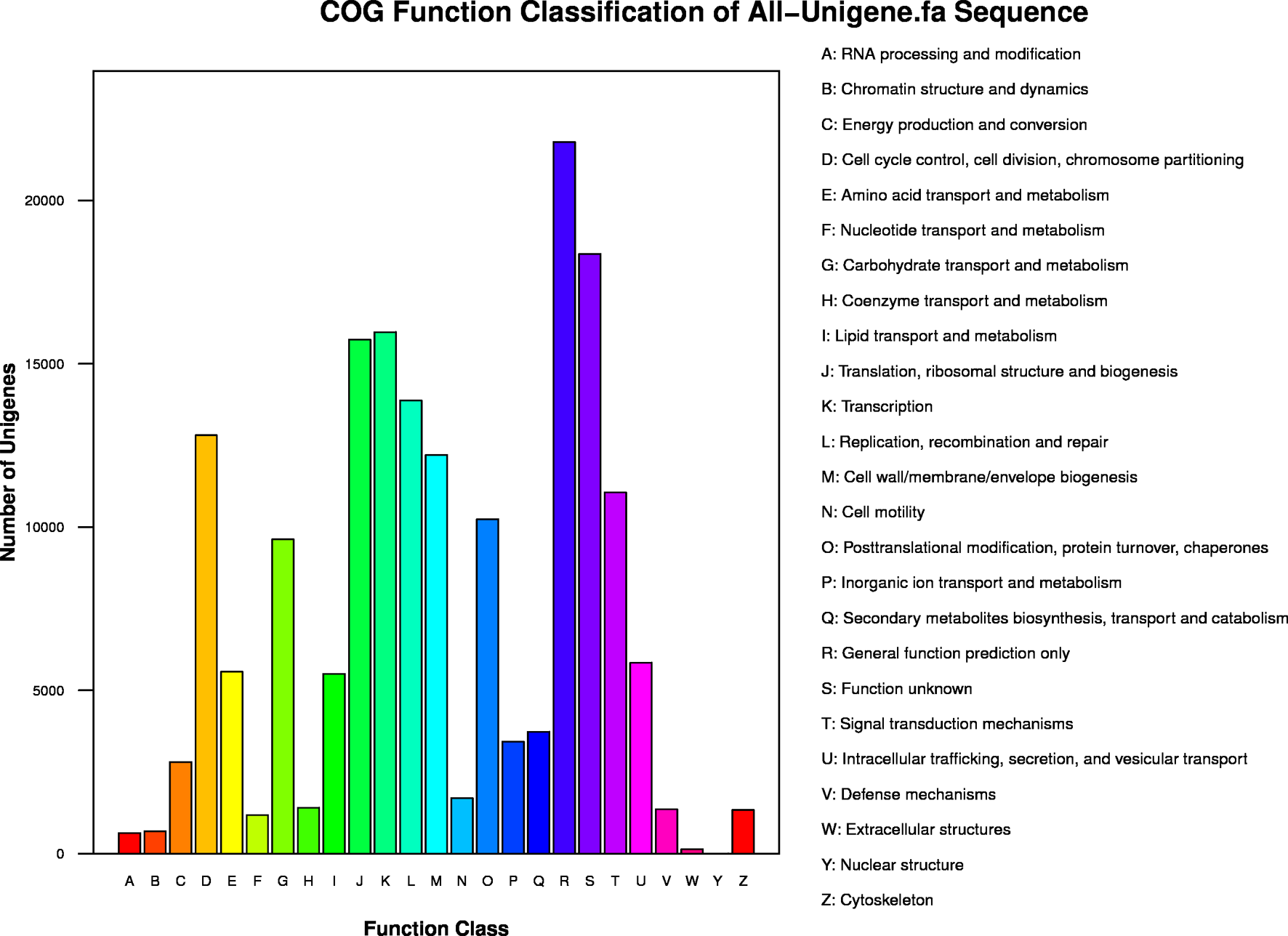

Supplement: S4 File — A total of 50,864 putative proteins showing significant homology to those in the COG database were functionally classified into 25 molecular families. (TIF) [file pone.0171451.s005.tif]

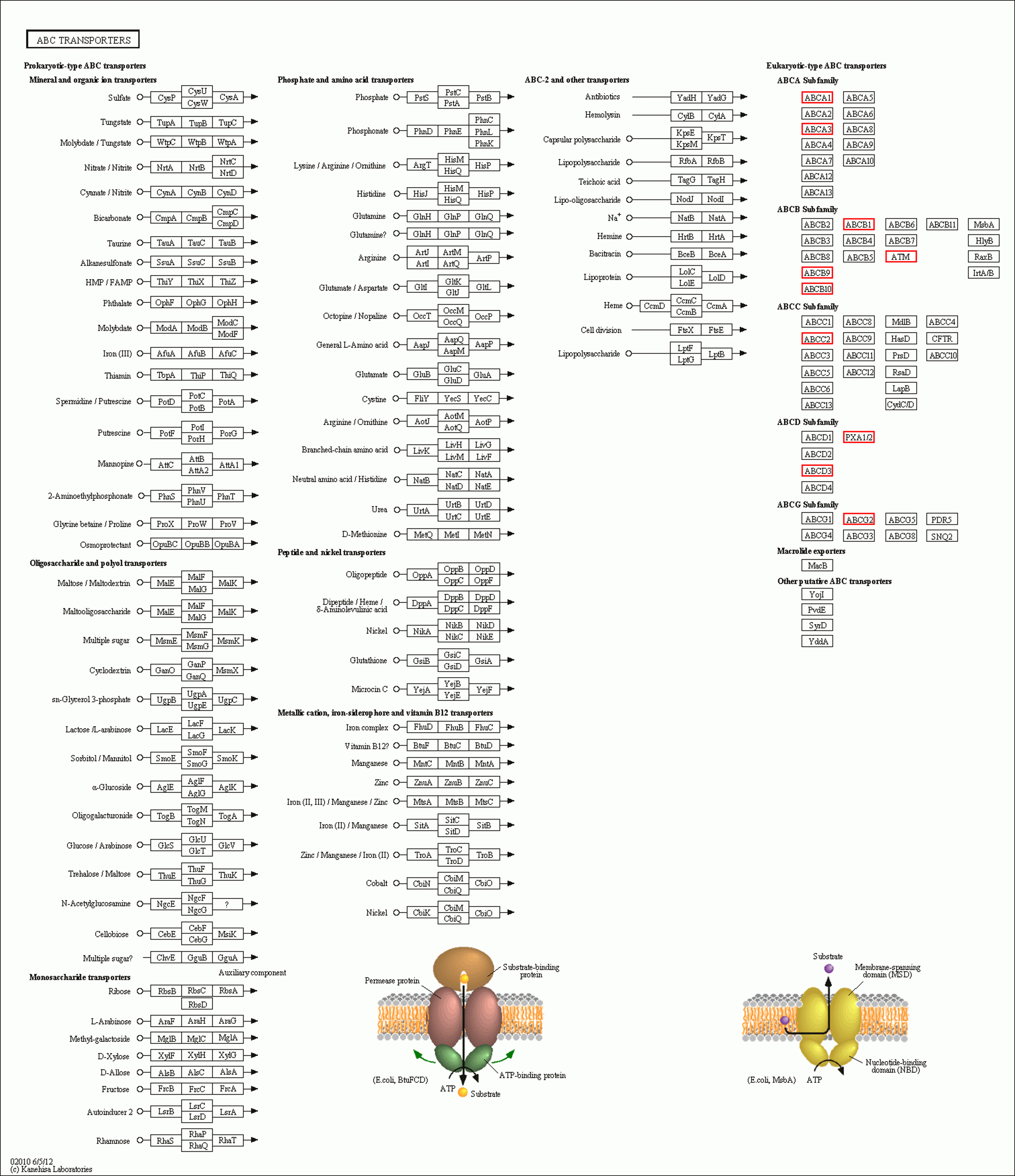

Supplement: S6 File — Map displays selected steps from KEGG pathways map02010 ‘ABC transporters’. Colors indicate significant expression, respective metabolite content ratios between salt stressed and normal conditions, red indicates higher relative levels in salt stressed samples, green indicates lower relative levels under salt stress. (TIF) [file pone.0171451.s007.tif]
